# A new criterion for defining tunnel portal failure using the strength reduction method

**DOI:** 10.1371/journal.pone.0345953

**Published:** 2026-04-09

**Authors:** Chengya Hua, Hongzhou Zhang, Chenguang Song, Leihua Yao

**Affiliations:** 1 School of Architecture and Civil Engineering, Langfang Normal University, Langfang, China; 2 School of Engineering and Technology, China University of Geosciences Beijing, Beijing, China; 3 China Institute of Geo-Environmental Monitoring (Guide Center of Prevention Technology for Geo-hazards, MNR) Beijing, Beijing, China; Henan Polytechnic University, CHINA

## Abstract

Recently, the application of the strength reduction method (SRM) to stability analysis of tunnel portals has become a trend. The key to employing the SRM lies in selecting an appropriate failure criterion. It is analyzed that the application characteristics of traditional criteria. Additionally, it is proposed that a new failure criterion—the variational criterion. Based on the numerical models, the effectiveness of the aforementioned work is validated. The results show that the displacement mutation at characteristic points (Criterion Ⅰ) is cumbersome to apply and involves a substantial workload. The plastic zone penetration (Criterion Ⅱ) lacks quantitative and clear standards. The calculation program non-convergence (Criterion Ⅲ) lacks a mechanical explanation. The energy mutation (Criterion Ⅳ) can effectively reflect the failure mechanism of the model. But it requires considerable computational effort. The variational criterion addresses these shortcomings while providing results with a relative error of no more than 1.6% compared to other criteria. Moreover, this applicability and accuracy are largely unaffected by mesh densities, geometric dimensions, strength reduction factor intervals, mechanical parameters, and convergence criteria. The variational criterion offers a comprehensive indicator—the variational value, and employs a clear discrimination method—judging the sign of the variational value. This criterion can provide a new reference for failure discrimination in tunnel portals.

## 1 Introduction

Tunnel portal is a complex structure with the interaction between the slope and the tunnel [1–3]. The evaluation of tunnel portal stability is of great significance for ensuring construction safety and reducing project cost. Factor of safety (FOS) is widely recognized as an important index to evaluate the stability. The strength reduction method (SRM) is an effective method to calculate the FOS of a slope [4,5]. It has been widely used to calculate the FOS of a tunnel portal or tunnel-slope system [6–9,10].

The key to applying the SRM lies in the selected failure criterion. In other structures, the failure criteria for the SRM are commonly adopted from slopes. In slopes, there are mainly three kinds of generally used criteria: Criterion Ⅰ—displacement of monitoring points mutation [11,12], Criterion Ⅱ—plastic zone penetration [13,14], and Criterion Ⅲ—calculation program non-convergence [15,16]. Additionally, there is a recently proposed criterion. It is the Criterion Ⅳ- energy mutation [17,18]. Based on this criterion, numerous scholars have conducted extensive research in both theoretical and applied aspects [9,19–23,24]. As is noted, the four types of criteria have their respective limitations [25–27]. In terms of Criterion Ⅰ, it is difficult to select node locations and displacement directions. In terms of Criterion Ⅱ, it lacks a clear standard for the penetration of plastic zone. In terms of Criterion Ⅲ, there is a lack of a certain standard for calculation program non-convergence. In terms of Criterion Ⅳ, significant workload is required, and human errors may occur.

However, when it refers to the tunnel portal, there are limited papers that analyze the applicability of existing failure criteria, or explores the establishment of a new failure criterion. Castillo and Luceno [28] explored the variational method in geotechnical engineering stability analysis. Leshchinsky and Huang [29] studied the applicability of the variational method for slope stability analysis. In view of the validity of the variational method and the convenience of the SRM, some researchers combined the two methods together [30,31]. According to the above research, a variational criterion is proposed to determine the failure of the tunnel portal in this paper. Meanwhile, the advantages and disadvantages of some existing failure criteria are analyzed. In Section 2, the SRM and four kinds of generally used failure criteria are introduced. The variational criterion is proposed. Specially, the formulas for calculating the variational value are derived. The program for calculating the variational value is written in FISH language. Based on the variational values, the procedure for judging model stability is presented. In Section 3, numerical models of tunnel portals are established. It is verified that the applicability of the proposed variational criterion. In Section 4, The mesh densities, geometric dimensions, strength reduction factor intervals, mechanical parameters, and convergence criteria are adjusted. It is verified that the generality of the proposed variational criterion. In Section 5, an engineering case of the slope-tunnel combination with heterogeneous soil layers is analyzed. It is verified that the engineering application value of the proposed variational criterion. In Section 6, discussions are made. In Section 7, conclusions are drawn.

## 2 Theories

### 2.1 Strength reduction method

A numerical model of the tunnel portal is established in the FLAC^3D^ software. The effective cohesion and effective friction angle of the model are continuously reduced to be substituted into the model to calculate until the model fails. The strength reduction factor (SRF) corresponding to the model failure is taken as the FOS. As shown in Eq. (1), the initial strength parameters (denoted as $c_{\text{0}}$ and $\varphi_{\text{0}}$) are divided by the SRF (denoted as k) to obtain the new strength parameters (denoted as ${\text{c}}^{\prime }$ and $\varphi^{\prime }$).


$$\left\{ \begin{array}{c} @lc^{\prime }=\frac{c_{\text{0}}}{k}\;\\ \varphi^{\prime }={\text{tan}}^{-\text{1}}(\frac{tan\varphi_{\text{0}}}{k}) \end{array}\right.\;$$ (1)

### 2.2 Four kinds of generally used failure criteria

(i) Criterion Ⅰ. Displacements of key nodes are monitored and curves between displacements and SRFs are plotted. The SRF corresponding to the point of abrupt change on the curve is determined as the FOS. (ii) Criterion Ⅱ. It is observed that the change of plastic elements of the model with the SRF increasing. The SRF corresponding to the plastic zone penetration is determined as the FOS. (iii) Criterion Ⅲ. The SRF corresponding to calculation program non-convergence is determined as the FOS. (iv) Criterion Ⅳ. Values of elastic or plastic zone energy of the model are calculated and curves between energy values and SRFs are plotted. The SRF corresponding to point of abrupt change on the curve is determined as the FOS. Relative calculation is shown in Eq. (2). Based on these formulas, a computational program written in the FISH language is developed in the FLAC^3D^ software to achieve the calculation of energy values.


$$\left\{ \begin{array}{c} \overset{\left(e\right)}{\underset{s}{E}}=\sum_{i=\text{1}}^{m}\overset{\left(e\right)}{\underset{i}{E}}=\sum_{i=\text{1}}^{m}\left[\sum_{j=\text{1}}^{x}\left(\overset{\left(ej\right)}{\underset{kl}{\sigma }}+\frac{\text{1}}{\text{2}}\Delta \overset{\left(ej\right)}{\underset{kl}{\sigma }}\right)\Delta \overset{\left(ej\right)}{\underset{kl}{\varepsilon }}\right]\left[v_{i}\right]\\ \overset{\left(p\right)}{\underset{s}{E}}=\sum_{i=\text{1}}^{n}\overset{\left(p\right)}{\underset{i}{E}}=\sum_{i=\text{1}}^{n}\left[\sum_{j=\text{1}}^{y}\left(\overset{\left(pj\right)}{\underset{kl}{\sigma }}+\frac{\text{1}}{\text{2}}\Delta \overset{\left(pj\right)}{\underset{kl}{\sigma }}\right)\Delta \overset{\left(pj\right)}{\underset{kl}{\varepsilon }}\right]\left[v_{i}\right] \end{array}\right.\;$$ (2)

Where, $\overset{\left(e\right)}{\underset{s}{E}}$ is the elastic zone energy of the model; $\overset{\left(e\right)}{\underset{i}{E}}$ is the total strain energy of some elastic element in the model; m is the total number of elements in the model that are currently in an elastic state; For a given element currently in an elastic state, $\overset{\left(ej\right)}{\underset{kl}{\sigma }}$, $\Delta \overset{\left(ej\right)}{\underset{kl}{\sigma }}$ and $\Delta \overset{\left(ej\right)}{\underset{kl}{\varepsilon }}$ represent the stress tensor, the stress increment tensor, and the strain increment tensor at the j−th time step during the finite difference calculation, respectively; x represents the total number of time steps required for the finite difference calculation. $\overset{\left(p\right)}{\underset{s}{E}}$ is the plastic zone energy of the model; $\overset{\left(p\right)}{\underset{i}{E}}$ is the total strain energy of some plastic element in the model; n is the total number of elements in the model that are currently in a plastic state; For a given element currently in a plastic state, $\overset{\left(pj\right)}{\underset{kl}{\sigma }}$, $\Delta \overset{\left(pj\right)}{\underset{kl}{\sigma }}$ and $\Delta \overset{\left(pj\right)}{\underset{kl}{\varepsilon }}$ represent the stress tensor, the stress increment tensor, and the strain increment tensor at the j−th time step during the finite difference calculation, respectively; y represents the total number of time steps required for the finite difference calculation. $v_{i}$ represent the volume of the i−th element of the model.

### 2.3 The proposed variational criterion

For the numerical model of a tunnel portal in FLAC^3D^, the algebraic sum of internal work (denoted as U) and external work (denoted as W) is the total potential energy (denoted as $E_{t}$). Here, the load condition is preliminarily defined as gravity loading only, while the boundaries are assumed to be non-working boundaries. Small virtual deformations satisfying the boundary conditions are applied to produce variation. When the model is in a steady state, according to the principle of minimum potential energy, $E_{t}$ takes the local minimum. At the same time, the second-order variational value of $E_{t}$ is greater than zero. Conversely, when the model is in a critical state of instability, $E_{t}$ takes the local maximum, and the second-order variational value of $E_{t}$ is less than zero. Relevant formulas are derived as Eqs. (3)-(4).


$$E_{t}=U-W$$ (3)

$$\delta E_{t}=\delta U-\delta W=\int \left[U\left(\overset{\text{0}}{\underset{ij}{\varepsilon }}+\delta \varepsilon_{ij}\right)-U\left(\overset{\text{0}}{\underset{ij}{\varepsilon }}\right)\right]d\Omega -\left\{\int \left[{\overset{―}{f}}_{i}\left(\overset{\text{0}}{\underset{i}{u}}+\delta u_{i}\right)-{\overset{―}{f}}_{i}\left(\overset{\text{0}}{\underset{i}{u}}\right)\right]d\Omega -\int \left[{\overset{―}{T}}_{i}\left(\overset{\text{0}}{\underset{i}{u}}+\delta u_{i}\right)-{\overset{―}{T}}_{i}\left(\overset{\text{0}}{\underset{i}{u}}\right)\right]dS\right\}$$ (4)

Where, $U,\;{\overset{―}{f}}_{i},\;{\overset{―}{T}}_{i}$ represent the strain energy, the body force and the surface force, respectively; $\overset{\text{0}}{\underset{ij}{\varepsilon }},\;\overset{\text{0}}{\underset{i}{u}}$ represent the initial strain tensor and the displacement tensor, respectively; $\delta \varepsilon_{ij},\;\delta u_{i}$ represent the first-order variational values of the strain tensor and the displacement tensor, respectively; S, Ω represent the area of model boundaries and the volume of model body, respectively.

When $U,\;{\overset{―}{f}}_{i},\;{\overset{―}{T}}_{i}$ are expanded according to Taylor’s series and the second-order minute quantity is ignored, the first-order variational value of $E_{t}$ can be expressed as Eq. (5).


$$\delta E_{t}=\int \overset{\text{0}}{\underset{ij}{\sigma }}\delta \varepsilon_{ij}d\Omega -\left[\int {\overset{―}{f}}_{i}\left(\delta u_{i}\right)d\Omega +\int {\overset{―}{T}}_{i}\left(\delta u_{i}\right)dS\right]$$ (5)

Where, $\overset{\text{0}}{\underset{ij}{\sigma }}$ represents the initial stress tensor.

For a certain model, ${\overset{―}{f}}_{i}$ is constant and the work done by ${\overset{―}{T}}_{i}$ is zero. Therefore, the formula for calculating the second-order variational value of $E_{t}$ can be expressed as Eq. (6).


$$\delta^{\text{2}}E_{t}=\int \delta \sigma_{ij}\delta \varepsilon_{ij}d\Omega$$ (6)

Where, $\delta \sigma_{ij}$ is the first-order variational value of the stress tensor.

The model stability is only related to the sign of the total variational value. It has nothing to do with the size of the total variational value. In order to unify the order of the magnitude of the total variational value of different models, the average variational value is defined. Hereafter, the average variational value is referred to as the mentioned variational value. The average variational value can be calculated as Eq. (7).


$$v_{v}=\frac{\int \delta \sigma_{ij}\delta \varepsilon_{ij}d\Omega }{\int d\Omega }$$ (7)

**Fig 1:**
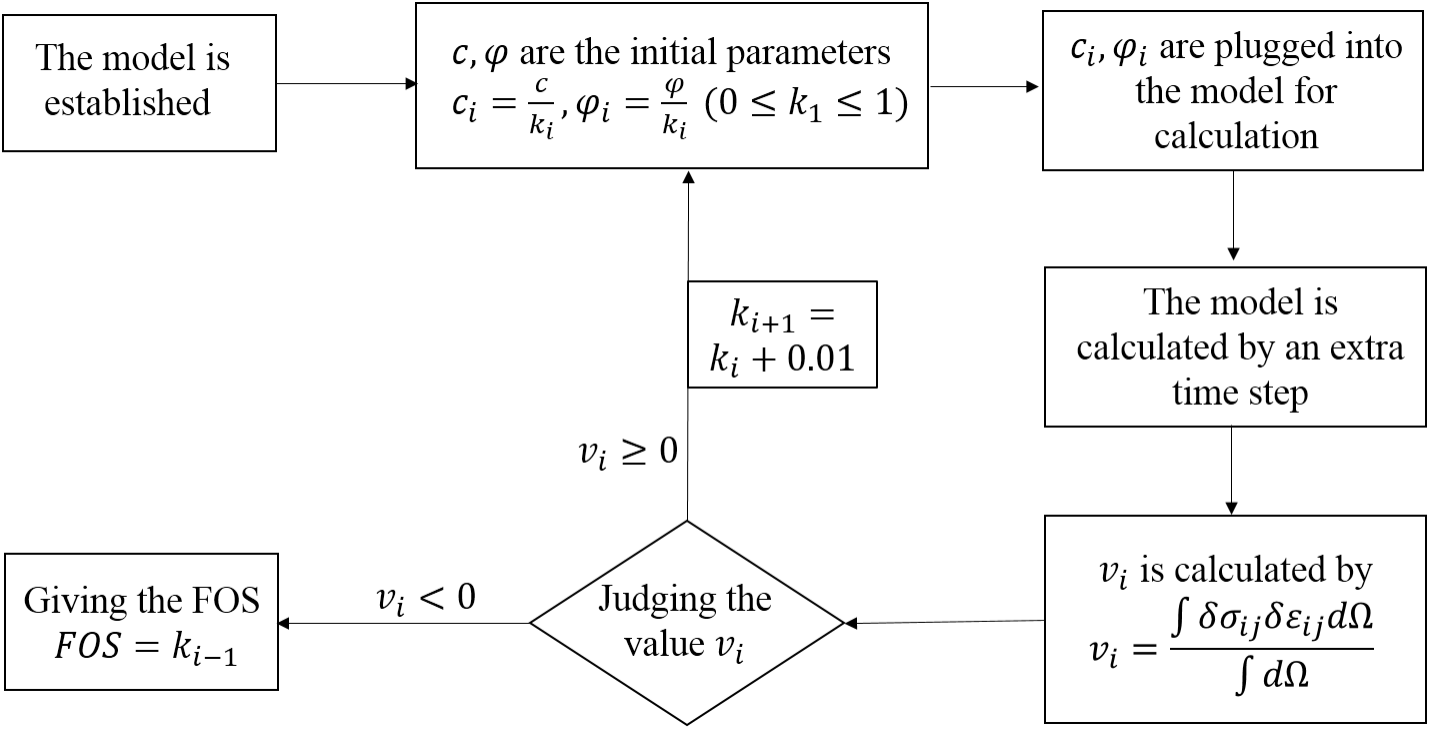
Flow chart of the application of the variational criterion.

With the help of FISH language, the program to calculate the variational value can be written. The calculation method is as follows. When the calculation is finished under a certain SRF, the stress and strain values of each element are restored and saved. Then the model is calculated with one more time step and the stress and strain values of each element are restored and saved again. Differences obtained by subtracting the former from the latter are taken as the first-order stress and strain variational values of the element. Finally, the variational value of the model is calculated according to the Eqs. (2)–(7). As shown in Fig 1, the process for applying the variational criterion is presented.

## 3 Verification

**Fig 2:**
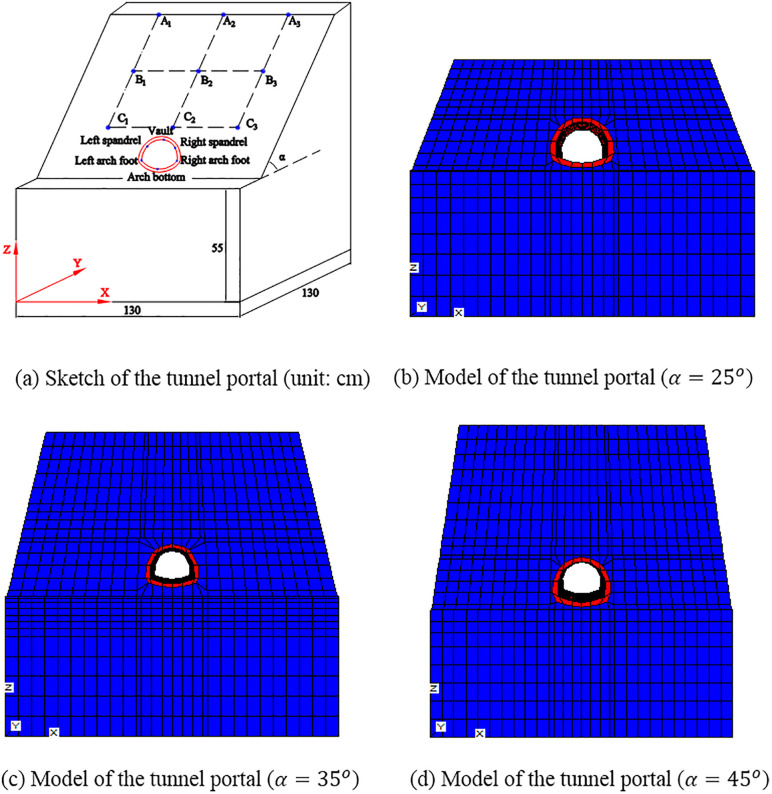
Sketch and numerical model of the tunnel portal.

As shown in Fig 2, according to the physical models in the experiment [32], three numerical models of tunnel portals with different slope angles are established. The slope angles (denoted as α) of the three models are ${\text{2}\text{5}}^{\text{0}},\;{\text{3}\text{5}}^{\text{0}}$ and ${\text{4}\text{5}}^{\text{0}}$, respectively. The geometric sizes, materials, locations of monitoring nodes and boundary conditions are the same. The model has a length of 130 cm in both the X and Y directions. The platform near the tunnel entrance is situated 55 cm above the base of the model. The tunnel has a width of 22 cm and a height of 17.54 cm. The lining has a thickness of 2 cm. The soil is with an elastic modulus of 30.1 MPa, a Poisson’s ratio of 0.4, a unit weight of $\text{2}\text{0}\;kN/m^{\text{3}}$, a cohesion of 1.428 kPa, and an internal friction angle of ${\text{2}\text{2}.\text{3}}^{\text{0}}$. The soil material is ideally elastoplastic and obeys Mohr-Coulomb failure criterion. The lining is with an elastic modulus of 0.58 MPa, a Poisson’s ratio of 0.2, and a unit weight of $\text{2}\text{4}.\text{2}\;kN/m^{\text{3}}$. The lining material is ideally elastic. The monitoring nodes are set on the slope face and the tunnel lining. The boundary conditions are: as shown in Fig 2a, on the planes at X = 0 cm and X = 130 cm, displacements in the X-direction are constrained; on the planes at Y = 0 cm and Y = 130 cm, displacements in the Y-direction are constrained; on the plane at Z = 0 cm, displacements in all three directions (X, Y, and Z) are fixed; the remaining surfaces of the model are set as free surfaces. As demonstrated in Fig 2b–d, the models are established in the FLAC^3D^ software. When $\alpha ={\text{2}\text{5}}^{\text{0}}$, the model contains 5656 elements and 6602 joints. When $\alpha ={\text{3}\text{5}}^{\text{0}}$, the model contains 9492 elements and 10701 joints. When $\alpha ={\text{4}\text{5}}^{\text{0}}$, the model contains 8876 elements and 10117 joints.

### 3.1 Four kinds of failure criteria

(1) Criterion Ⅰ

**Fig 3:**
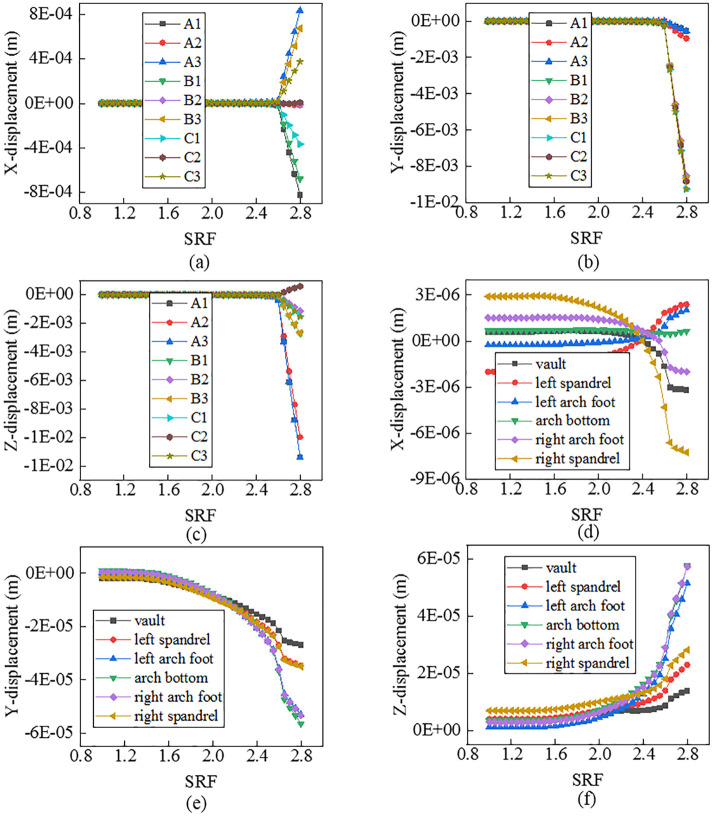
Curves between displacements and SRFs for nodes when $\alpha =25^{0}$.

**Fig 4:**
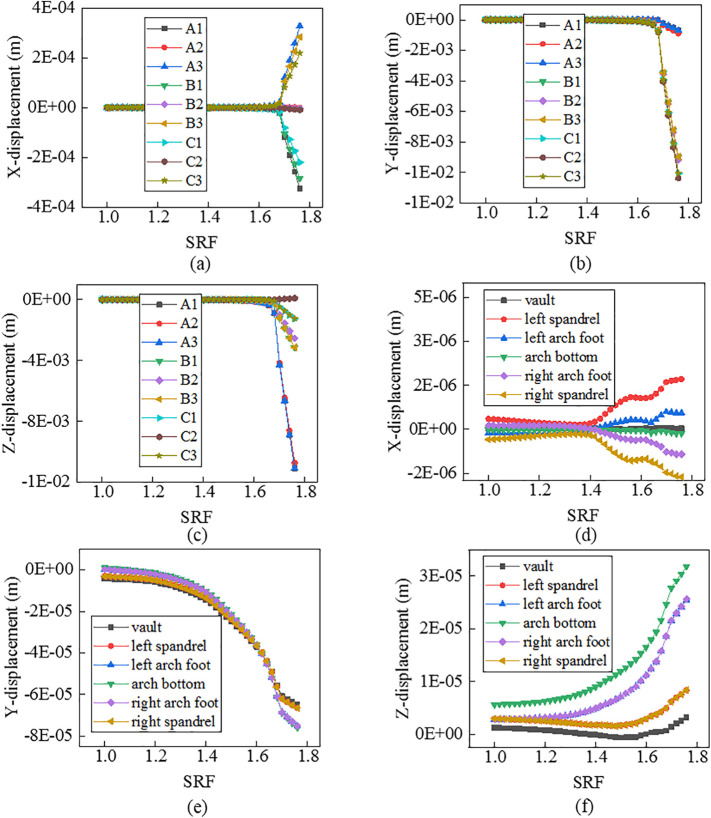
Curves between displacements and SRFs for nodes when $\alpha =35^{0}$.

**Fig 5:**
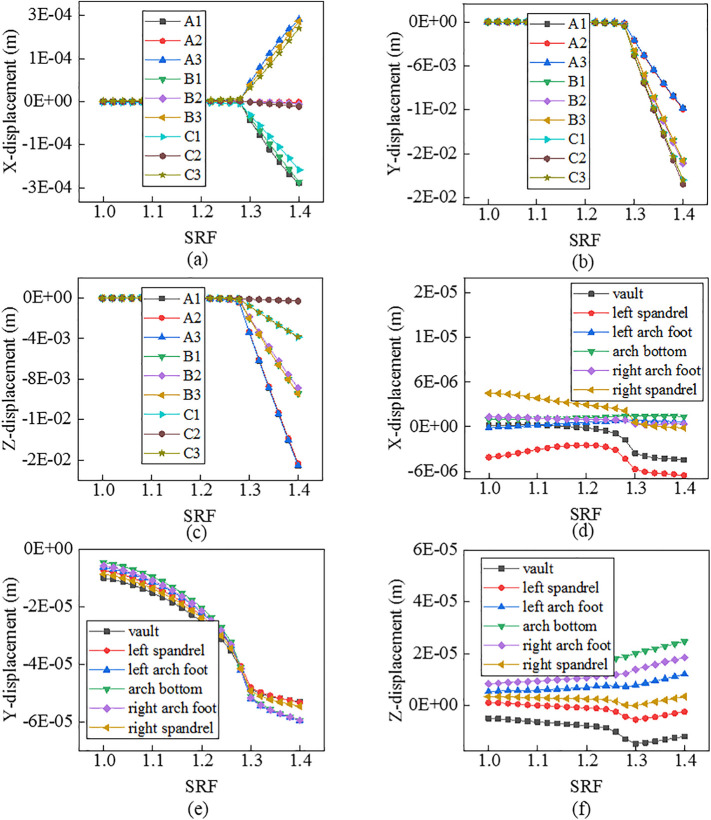
Curves between displacements and SRFs for nodes when $\alpha =45^{0}$.

As shown in Figs 3–5, there is a consistent rule for the three models. Curves for nodes on the slope face and the tunnel lining demonstrate different characteristics. For the former, there is an obvious mutation. For the latter, it has no evident mutation.

For nodes on the slope face, shapes of the curves vary with displacement directions and node locations. In terms of displacement in the X direction, curves for nodes on line A_2_-B_2_-C_2_ have no obvious mutation, while those on lines A_1_-B_1_-C_1_ and A_3_-B_3_-C_3_ have an evident mutation. Specifically, the mutation of curves for nodes on the top of slope (A_1_, A_3_) is the most violent, that on the middle of slope (B_1_, B_3_) is the second, and that on the bottom of slope (C_1_, C_3_) is the weakest. It means that the top of slope is a weak area for displacements in the X direction. In terms of displacements in the Y direction, curves for all nodes have an obvious mutation. Specifically, the mutation of curves for nodes on lines C_1_-C_2_-C_3_ and B_1_-B_2_-B_3_ is the more violent than that on line A_1_-A_2_-A_3_. It means that the middle and bottom of slope are weak areas for displacements in the Y direction. In terms of displacements in the Z direction, curves for all nodes have an evident mutation. Specifically, the mutation of curves for nodes on line A_1_-A_2_-A_3_ is the most violent, that on line B_1_-B_2_-B_3_ is the second, and that on line C_1_-C_1_-C_3_ is the weakest. It means that the top of slope is a weak area for displacements in the Z direction. For nodes on the tunnel lining, shapes of the curves also vary with displacement directions and node locations. In terms of displacements in the X direction, displacements of the spandrel, the arch foot, the vault and the arch bottom decreases in turn, and the mutation of curves for those nodes also weakens in turn. In terms of displacements in the Y direction, displacements of all nodes are close to each other, and the mutation of curves for all nodes is not obvious. In terms of displacements in the Z direction, when the slope angle varies, curves for all nodes do not show coincident mutation. Specifically, when the slope angle is ${\text{2}\text{5}}^{o}$ or ${\text{3}\text{5}}^{o}$, the mutation of curves for nodes on the arch foot and arch bottom is the most violent, that on the spandrel is the second, and that on the vault is the weakest. When the slope angle is ${\text{4}\text{5}}^{o}$, there is no evident mutation for curves for all nodes.

In summary, compared to tunnel linings, nodes on slope faces offer greater monitoring value. However, substantial effort is required to obtain a reliable safety factor. Based on the curves exhibiting distinct points of abrupt change, FOSs are confirmed as 2.62, 1.68 and 1.28 when slope angles are ${\text{25}}^{\text{0}}$, ${\text{35}}^{\text{0}}$ and ${\text{45}}^{\text{0}}$, respectively.

(2) Criterion Ⅱ

**Fig 6:**
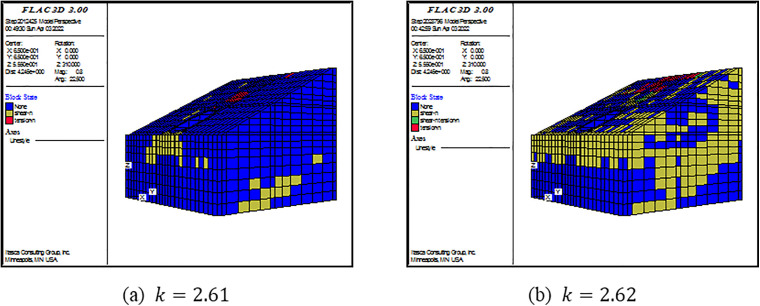
Pictures of failure elements under different SRFs when $\alpha =25^{0}$.

**Fig 7:**
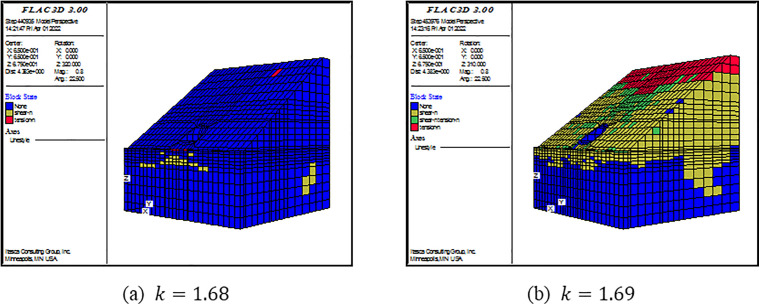
Pictures of failure elements under different SRFs when $\alpha =35^{0}$.

**Fig 8:**
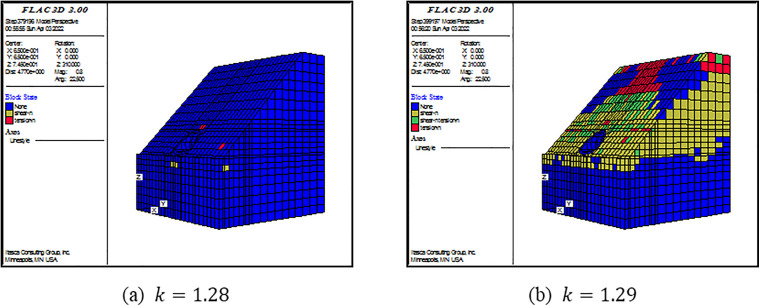
Pictures of failure elements under different SRFs when $\alpha =45^{0}$.

As shown in Figs 6–8, there is also a consistent rule for the three models. When the SRF is some certain value, there are not plenty of yielding elements in the model. When the SRF exceeds the certain value, a large number of yielding elements are generated from the top to the foot of slope. Specially, the tensile failure is dominant at the top of slope, the simultaneous tensile and shear failure are dominant at the middle of slope, and the shear failure is dominant at the foot of slope. As revealed by this perspective, the failure occurring is consistent with the displacement curve mutation. Under this criterion, FOSs are confirmed as 2.61, 1.68 and 1.28 when slope angles are ${\text{25}}^{\text{0}}$, ${\text{35}}^{\text{0}}$ and ${\text{45}}^{\text{0}}$, respectively.

(3) Criterion Ⅲ

The built-in FOS command in FLAC^3D^ can directly give the FOS of a model. The essence of this command is to apply SRM based on the criterion of calculation program non-convergence. However, there are two drawbacks: (1) the command cannot calculate the model with the elastic material; (2) the default range for the reduction factor in this command is 0–64; it results in a lengthy computation time. For convenience and to save computational time, a self-programed command is written in FISH language. The main points of the command are as follows. First, the ratio of maximal unbalance force and the number of limit time step are set as $\text{1}.\text{0}\times {\text{1}\text{0}}^{-\text{5}}$ and 10000, respectively. Second, a range of the SRF is self-defined. Here, it is the 0–5. Using the bisection method, the SRF is iteratively adjusted. The computation is finished when the difference between two consecutive SRFs became less than 0.02. Finally, the output SRF is defined as the FOS of the model. To verify the self-programed command, the lining is set as the elastoplastic material with large effective cohesion and friction angle. The material obeys Mohr-Coulomb failure criterion. Then, the FOS command is applied.

**Fig 9:**
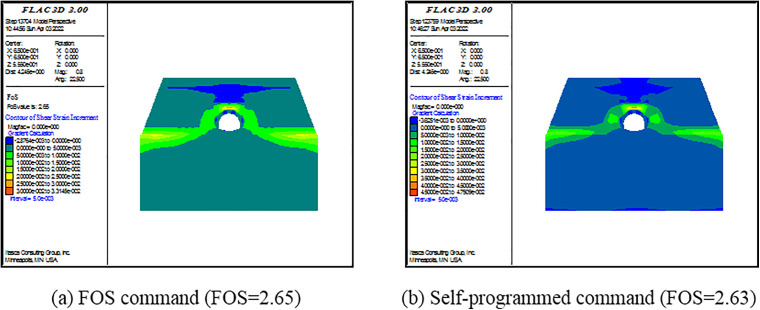
Pictures of the shear strain increment of the model under different commands when $\alpha =25^{0}$.

**Fig 10:**
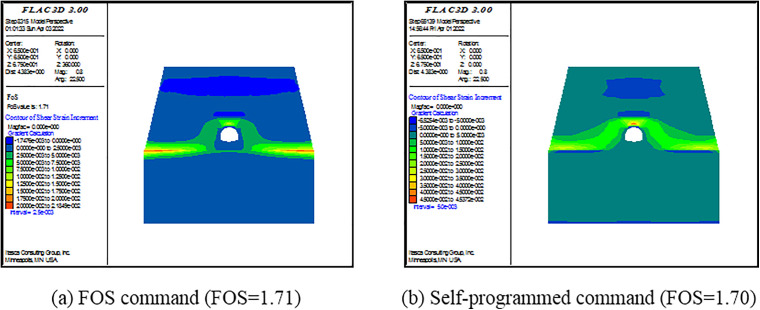
Pictures of the shear strain increment of the model under different commands when $\alpha =35^{0}$.

**Fig 11:**
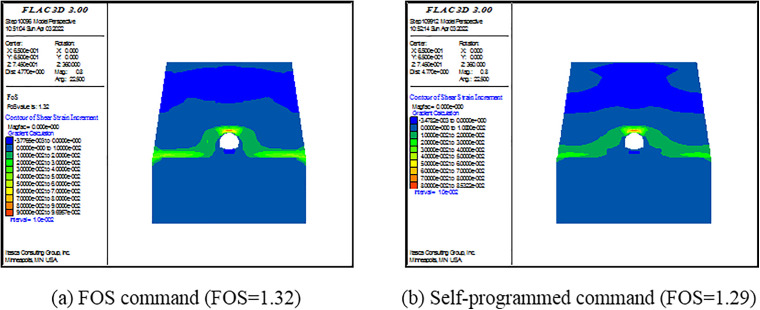
Pictures of the shear strain increment of the model under different commands when $\alpha =45^{0}$.

FOSs calculated by the self-programed command are compared with those calculated by the FOS command. As shown in Figs 9–11, FOSs calculated by the self-programed command are slightly smaller than those calculated by the FOS command. Specially, relative errors between the two are 0.8%, 0.6% and 2.3% when slope angles are ${\text{2}\text{5}}^{o}$, ${\text{3}\text{5}}^{o}$ and ${\text{4}\text{5}}^{o}$, respectively. It is demonstrated that the self-programed command is applicable and accurate. Hereafter, both the self-programed command and the FOS command are collectively referred to as the FOS command. Under this criterion, FOSs are confirmed as 2.63, 1.70 and 1.29 when slope angles are ${\text{25}}^{\text{0}}$, ${\text{35}}^{\text{0}}$ and ${\text{45}}^{\text{0}}$, respectively.

(4) Criterion Ⅳ

**Fig 12:**
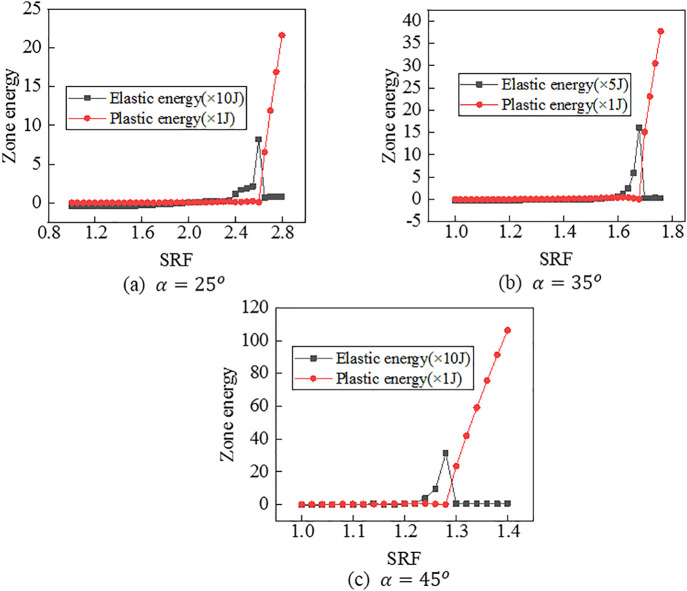
Curves between elastic and plastic zone energy values and SRFs.

According to the Eq. (2), it is written a program in FISH language that is used to calculate the elastic or plastic zone energy of the model under a certain SRF. Then, values of elastic or plastic zone energy of the model under different SRFs can be obtained. As shown in Fig 12, there is a consistent rule for the three models. In terms of the elastic zone energy, the value increases when the SRF is less than some certain value, reaches the top when the SRF is equal to the certain value, and decreases when the SRF exceeds the certain value. In terms of the plastic zone energy, the value increases slowly when the SRF is less than some certain value, but increases rapidly when the SRF exceeds the certain value. The change of the energy curve accurately captures the evolution process of the tunnel portal from stability to failure. Under this criterion, FOSs are confirmed as 2.60, 1.68 and 1.28 when slope angles are ${\text{25}}^{\text{0}}$, ${\text{35}}^{\text{0}}$and ${\text{45}}^{\text{0}}$, respectively.

### 3.2 The proposed variational criterion

As shown in Tables 1–3, there is a consistent rule for the three models. When the SRF is less than or equal to some certain value, the variational value is positive. When the SRF exceeds the certain value, the variational value becomes negative for the first time. The change of the sign of the variational value is consistent with the process of plasticity developing and failure occurring. Under this criterion, FOSs are confirmed as 2.64, 1.69 and 1.30 when slope angles are ${\text{2}\text{5}}^{\text{0}}$, ${\text{3}\text{5}}^{\text{0}}$and ${\text{4}\text{5}}^{\text{0}}$, respectively.

**Table 1:** Variational values and SRFs when $\alpha =25^{0}$.

SRF	variational value	State	SRF	variational value	State	SRF	variational value	State
1.00	6.88E-12	stable	2.60	8.93E-12	stable	2.64	1.24E-08	stable
1.50	6.15E-12	stable	2.61	2.39E-11	stable	2.65	−3.00E-08	unstable
2.00	7.30E-12	stable	2.62	8.16E-12	stable			
2.50	1.03E-11	stable	2.63	8.84E-10	stable			

**Table 2:** Variational values and SRFs when $\alpha =35^{\text{0}}$.

SRF	Variational value	State	SRF	Variational value	State	SRF	Variational value	State
1.00	1.23E-11	stable	1.50	1.65E-11	stable	1.68	1.70E-11	stable
1.10	1.45E-11	stable	1.60	2.04E-11	stable	1.69	1.13E-10	stable
1.20	1.35E-11	stable	1.65	1.92E-11	stable	1.70	−3.57E-09	unstable
1.30	1.56E-11	stable	1.66	1.95E-11	stable			
1.40	1.79E-11	stable	1.67	2.05E-11	stable			

**Table 3:** Variational values and SRFs when $\alpha =45^{0}$.

SRF	Variational value	State	SRF	Variational value	State	SRF	Variational value	State
1.00	1.77E-11	stable	1.15	1.77E-11	stable	1.30	1.73E-08	stable
1.05	1.78E-11	stable	1.20	1.51E-11	stable	1.31	−8.24E-08	unstable
1.10	1.57E-11	stable	1.25	1.70E-11	stable			

### 3.3 Comparisons

As shown in Table 4, results of the variational criterion are close to those of other criteria. There are maximal values of absolute errors and relative errors of 0.04 and 1.6% between them, respectively. The small errors show that the proposed variational criterion is of good applicability and accuracy. Furthermore, it can be seen that there is a certain difference between results of the variational criterion and results of other criteria. It may be attributed to the following reasons. For Criterion Ⅰ, the location of the characteristic point and the choice of displacement direction have a significant impact on the determination of the FOS. Moreover, the identification of the displacement curve mutation relies on human judgment, introducing a certain subjective error. For Criterion Ⅱ, for complex structures such as the tunnel portal, there is a lack of a unified and clear standard for plastic zone connection. Instability can only be assumed based on the experience that the shape of the plastic zone is like the ‘Ω’, which also introduces a certain degree of ambiguity and human error. For Criterion Ⅲ, the FOS command defaults to standards based on a maximum unbalanced force ratio of $\text{1}.\text{0}\times {\text{1}\text{0}}^{-\text{5}}$ and a limit time step number of 200000 [33]. However, a clearer and more unified mechanical explanation for this standard is currently lacking. For Criterion Ⅳ, on one hand, the shape of the energy curve is affected by the interval of the selected SRF; on the other hand, the identification of the mutation point is subject to human influence, thus introducing a certain subjective error. For the variational criterion, the stability of the model is characterized by the variational value and the instability of model is determined by justifying the sign of the variational value. The variational value contains the stress-strain information of all elements in the model, making it highly comprehensive. The approach to determine instability is highly objective, effectively improving the shortcomings of other criteria. By the proposed variational criterion, a new reference is provided for other criteria.

**Table 4:** FOSs under different criteria.

Slope angle *α* (^o^)	Criteria	FOS	Absolute error (absolute value)	Relative error (absolute value)
25	Variational criterion (VC)	2.64	—	—
	Criterion Ⅰ	2.62	0.02(VC-Ⅰ)	0.8%((VC-Ⅰ)/Ⅰ)
	Criterion Ⅱ	2.61	0.03(VC-Ⅱ)	1.1%((VC-Ⅱ)/Ⅱ)
	Criterion Ⅲ	2.63	0.01(VC-Ⅲ)	0.4%((VC-Ⅲ)/Ⅲ)
	Criterion Ⅳ	2.60	0.04(VC-Ⅳ)	1.5%((VC-Ⅳ)/Ⅳ)
35	Variational criterion (VC)	1.69	—	—
	Criterion Ⅰ	1.68	0.01(VC-Ⅰ)	0.6%((VC-Ⅰ)/Ⅰ)
	Criterion Ⅱ	1.68	0.01(VC-Ⅱ)	0.6%((VC-Ⅱ)/Ⅱ)
	Criterion Ⅲ	1.70	0.01(VC-Ⅲ)	0.6%((VC-Ⅲ)/Ⅲ)
	Criterion Ⅳ	1.68	0.01(VC-Ⅳ)	0.6%((VC-Ⅳ)/Ⅳ)
45	Variational criterion (VC)	1.30	—	—
	Criterion Ⅰ	1.28	0.02(VC-Ⅰ)	1.6%((VC-Ⅰ)/Ⅰ)
	Criterion Ⅱ	1.28	0.02(VC-Ⅱ)	1.6%((VC-Ⅱ)/Ⅱ)
	Criterion Ⅲ	1.29	0.01(VC-Ⅲ)	0.8%((VC-Ⅲ)/Ⅲ)
	Criterion Ⅳ	1.28	0.02(VC-Ⅳ)	1.6%((VC-Ⅳ)/Ⅳ)

## 4 Analysis of influenced factors

In this section, the mesh densities, geometric dimensions, strength reduction factor intervals, mechanical parameters, and convergence criteria are adjusted. Results of the FOS command are compared with those of the variational criterion. By this way, it is demonstrated that the generality of the proposed variational criterion. Here, the mesh size is denoted as s, the tunnel span as W, the tunnel height as H, and the lining thickness as f.

### 4.1 Mesh densities

Based on the three models in the section 3, the mesh densities are changed. It is explored that the generality of the proposed variational criterion under different mesh densities. As shown in Table 5, results of the variational criterion are in close agreement with those of the FOS command, with an average relative error of only 0.7% between the two. It demonstrates that the applicability of the proposed variational criterion is not coincidental with respect to mesh densities.

**Table 5:** FOSs under different mesh densities.

*α*(^o^)	(Result of the FOS command, Result of the variational criterion)								
	*s*(m)								
	0.003	0.004	0.005	0.006	0.007	0.008	0.009	0.010	0.011
25	2.56, 2.55	2.63, 2.64	2.66, 2.65	2.71, 2.71	2.73, 2.72	2.81, 2.80	2.82, 2.85	2.85, 2.84	2.88, 2.88
35	1.66, 1.65	1.70, 1.71	1.72, 1.71	1.74, 1.76	1.76, 1.75	1.81, 1.82	1.83, 1.85	1.84, 1.89	1.87, 1.90
45	1.26, 1.25	1.27, 1.27	1.29, 1.32	1.31, 1.33	1.33, 1.33	1.37, 1.38	1.37, 1.37	1.40, 1.41	1.42, 1.44

### 4.2 Geometric dimensions

Based on the three models in the section 3, the geometric dimensions are changed. It is explored that the generality of the proposed variational criterion under different geometric dimensions. As shown in Table 6, results of the variational criterion are also in close agreement with those of the FOS command, with an average relative error of only 0.7% between the two. It indicates that the applicability of the proposed variational criterion is not coincidental with respect to geometric dimensions.

**Table 6:** FOSs under different geometric dimensions.

*W*(m)	*H*(m)	(Result of the FOS command, Result of the variational criterion)								
		*d*(m)								
		0.02			0.03			0.04		
		*α*(^o^)			*α*(^o^)			*α*(^o^)		
		25	35	45	25	35	45	25	35	45
0.14	0.10	2.55, 2.57	1.67, 1.67	1.26, 1.26	2.58, 2.56	1.67, 1.67	1.26, 1.26	2.62, 2.61	1.69, 1.68	1.28, 1.27
	0.14	2.60, 2.60	1.68, 1.68	1.28, 1.30	2.63, 2.63	1.71, 1.70	1.28, 1.28	2.67, 2.67	1.72, 1.75	1.30, 1.32
	0.1754	2.67, 2.67	1.72, 1.75	1.30, 1.29	2.69, 2.67	1.74, 1.72	1.30, 1.35	2.72, 2.73	1.75, 1.75	1.32, 1.31
0.18	0.10	2.56, 2.55	1.66, 1.65	1.26, 1.25	2.59, 2.60	1.68, 1.67	1.27, 1.28	2.62, 2.62	1.70, 1.69	1.28, 1.27
	0.14	2.61, 2.60	1.70, 1.69	1.28, 1.28	2.65, 2.64	1.71, 1.71	1.29, 1.28	2.67, 2.66	1.72, 1.75	1.30, 1.31
	0.1754	2.65, 2.63	1.71, 1.71	1.30, 1.34	2.68, 2.68	1.73, 1.76	1.30, 1.29	2.72, 2.77	1.75, 1.74	1.32, 1.31
0.22	0.10	2.58, 2.57	1.67, 1.71	1.26, 1.25	2.60, 2.60	1.68, 1.68	1.27, 1.28	2.62, 2.61	1.69, 1.71	1.28, 1.31
	0.14	2.62, 2.63	1.69, 1.69	1.28, 1.29	2.65, 2.64	1.71, 1.72	1.28, 1.29	2.67, 2.69	1.72, 1.77	1.30, 1.31
	0.1754	2.66, 2.65	1.72, 1.71	1.29, 1.32	2.69, 2.68	1.75, 1.76	1.31, 1.32	2.72, 2.70	1.75, 1.76	1.32, 1.31

### 4.3 Strength reduction factor intervals

It has been demonstrated previously that the applicability of the variational criterion is not a coincidence of mesh densities and geometric dimensions. To save computational time, some models in the section 4.1 are selected for conducting experiments on the randomness of non-strength reduction factor intervals. These models are with slope angles of 25°, 35°, and 45°, and mesh sizes of 0.009 m, 0.010 m, and 0.011 m, respectively. Based on these models, the strength reduction factor intervals are changed. It is explored that the generality of the proposed variational criterion under different strength reduction factor intervals. As shown in Table 7, results of the variational criterion with the strength reduction factor interval of 0.01 are in close agreement with those of the FOS command, with an average relative error of only 0.9% between the two. Results of the variational criterion with the strength reduction factor interval of 0.001 are in closer agreement with those of the FOS command, with an average relative error of only 0.3% between the two. It demonstrates that the applicability of the proposed variational criterion is not coincidental with respect to strength reduction factor intervals.

**Table 7:** FOSs under different strength reduction factor intervals.

*α*(^o^)	*s*(m)	Result of the FOS command	Result of the variational criterion	
			Strength reduction factor interval of 0.01	Strength reduction factor interval of 0.001
25	0.009	2.82	2.85	2.815
	0.010	2.85	2.84	2.840
	0.011	2.88	2.88	2.882
	0.009	1.83	1.85	1.826
35	0.010	1.84	1.89	1.834
	0.011	1.87	1.89	1.866
	0.009	1.37	1.37	1.371
45	0.010	1.40	1.41	1.410
	0.011	1.42	1.44	1.423

### 4.4 Mechanical parameters

As described above, the same models are selected from the section 4.1 for conducting experiments on the randomness of non-mechanical parameters. These models are also with slope angles of 25°, 35°, and 45°, and mesh sizes of 0.009 m, 0.010 m, and 0.011 m, respectively. Based on these models, the mechanical parameters are changed. It is explored that the generality of the proposed variational criterion under different mechanical parameters. As shown in Table 8, results of the variational criterion remain close to those of the FOS command, with an average relative error of 1.0% between the two. It indicates that the applicability of the proposed variational criterion is not coincidental with respect to mechanical parameters.

**Table 8:** FOSs under adjusted mechanical parameters.

*α*(^o^)	*s*(m)	(Result of the FOS command, Result of the variational criterion)			
		*c*(kPa)			
		1.000		1.428	
		*φ*(^o^)		*φ*(^o^)	
		10.0	22.3	10.0	22.3
25	0.009	(1.57,1.62)	(2.39,2.38)	(1.96,1.97)	(2.82,2.85)
	0.010	(1.59,1.63)	(2.41,2.42)	(1.99,1.99)	(2.85,2.84)
	0.011	(1.61,1.66)	(2.45,2.44)	(2.01,2.02)	(2.88,2.88)
	0.009	(1.01,1.01)	(1.56,1.56)	(1.25,1.26)	(1.83,1.85)
35	0.010	(1.02,1.01)	(1.57,1.56)	(1.26,1.25)	(1.84,1.89)
	0.011	(1.04,1.03)	(1.60,1.59)	(1.28,1.27)	(1.87,1.90)
	0.009	(0.75,0.75)	(1.17,1.19)	(0.93,0.94)	(1.37,1.37)
45	0.010	(0.78,0.77)	(1.20,1.19)	(0.95,0.95)	(1.40,1.41)
	0.011	(0.78,0.80)	(1.22,1.23)	(0.96,0.97)	(1.42,1.44)

### 4.5 Convergence criteria

As stated above, the same models are selected from the section 4.1 for conducting experiments on the randomness of non-convergence criteria. These models are also with slope angles of 25°, 35°, and 45°, and mesh sizes of 0.009 m, 0.010 m, and 0.011 m, respectively. Based on these models, the convergence criteria are changed. It is explored that the generality of the proposed variational criterion under different convergence criteria. As shown in Table 9, results of the variational criterion remain close to those of the FOS command, with an average relative error of 0.6% between the two. It indicates that the applicability of the proposed variational criterion is not coincidental with respect to convergence criteria.

**Table 9:** FOSs under adjusted convergence criteria.

*α* (^o^)	*s*(m)	Result of the FOS command	Result of the variational criterion			
			Ratio of the maximal unbalanced force			
			1.00E-05		1.00E-06	
			Number of the limit time step		Number of the limit time step	
			10000	30000	10000	30000
	0.009	2.82	2.85	2.83	2.82	2.83
25	0.010	2.85	2.84	2.83	2.86	2.84
	0.011	2.88	2.88	2.89	2.87	2.89
	0.009	1.83	1.85	1.82	1.83	1.84
35	0.010	1.84	1.89	1.83	1.86	1.83
	0.011	1.87	1.90	1.88	1.86	1.87
	0.009	1.37	1.37	1.36	1.37	1.36
45	0.010	1.40	1.41	1.40	1.46	1.41
	0.011	1.42	1.44	1.42	1.42	1.42

It is seen that under different mesh densities, geometric dimensions, strength reduction factor intervals, mechanical parameters, and convergence criteria, results of the variational criterion are close to those of the FOS command, with an average relative error not exceeding 1.0%. It indicates the generality of the proposed variational criterion.

## 5 Engineering application of the variational criterion

**Fig 13:**
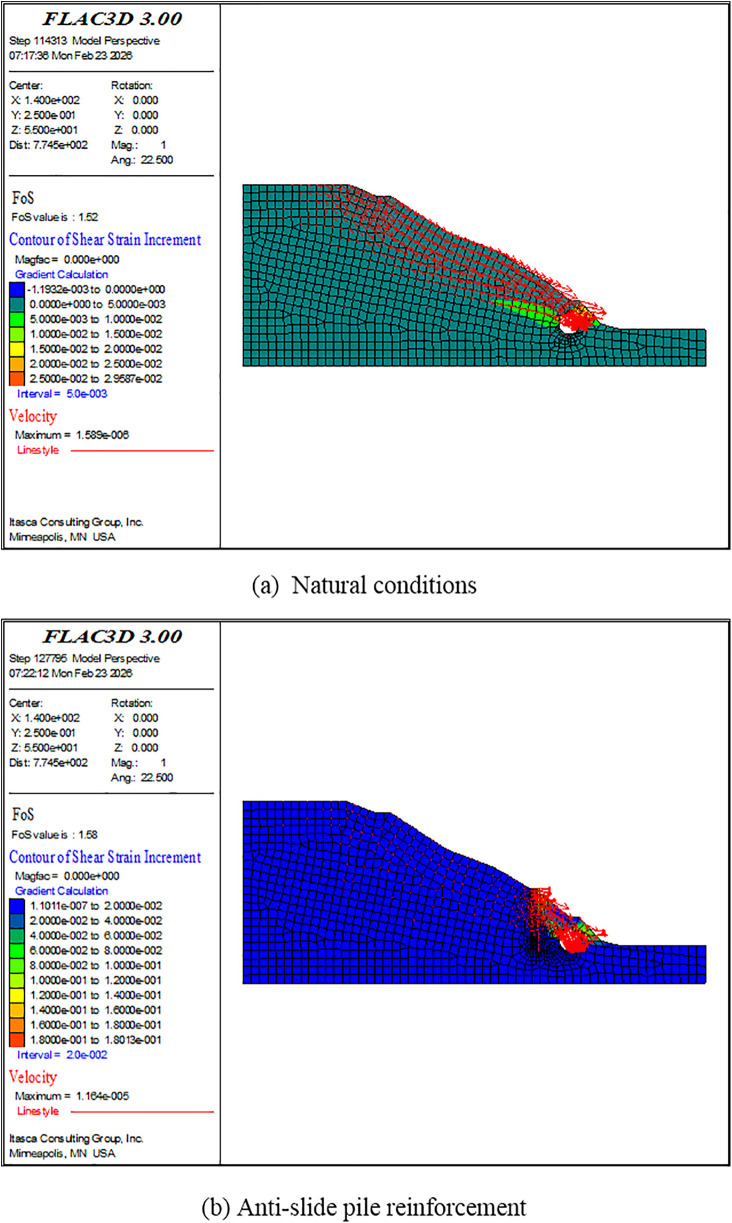
Nephograms of the slope-tunnel system engineering example when the failure occurs.

**Fig 14:**
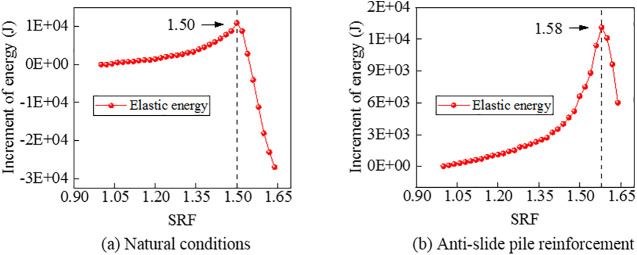
Curves between elastic energy increment values and SRFs for the slope-tunnel system engineering example. Note: the elastic energy is calculated by referencing the method in papers [17,18], and it yields more ideal results.

Referring to an engineering case of the slope-tunnel combination with heterogeneous soil layers [7], the validity of the variational criterion is verified. Specifically, two examples are employed: one is under natural conditions and the other is with the anti-slide pile reinforcement. First, the FOS command is applied. Results of the FOS command are taken as representative results of Criteria Ⅰ, Ⅱ, and Ⅲ. As illustrated in Fig 13, with the installation of anti-slide piles, the sliding of the slope on the left side of the tunnel is effectively resisted and the FOS is slightly increased. It is consistent with the conclusions of Zhang et al. [7]. Second, the elastic energy mutation criterion is applied. Results of the elastic energy mutation criterion are taken as representative results of Criterion Ⅳ. As shown in Fig 14, under a specific SRF for the two conditions, a significant and sudden reduction occurs in the increment of elastic energy of the model. Third, the variational criterion is applied. As shown in Tables 10 and 11, under a specific SRF for the two conditions, the variational values transition from positive to negative, indicating the occurrence of instability. Finally, different results are summarized in Table 12. As shown in Table 12, results of the variational criterion are close to results of other criteria and Zhang et al. [7]. This demonstrates the reliability of the numerical modeling in this paper and the engineering applicability of the proposed variational criterion.

**Table 10:** Variational values and SRFs when the slope-tunnel system is under natural conditions.

SRF	Variational value	State	SRF	Variational value	State	SRF	Variational value	State
1.00	6.30E-10	stable	1.20	6.03E-10	stable	1.40	4.68E-10	stable
1.05	4.66E-10	stable	1.25	3.98E-10	stable	1.45	3.06E-10	stable
1.10	4.72E-10	stable	1.30	6.02E-10	stable	1.50	1.22E-09	stable
1.15	5.65E-10	stable	1.35	4.06E-10	stable	1.51	−2.61E-09	unstable

**Table 11:** Variational values and SRFs when the slope-tunnel system is with the anti-slide pile reinforcement.

SRF	Variational value	State	SRF	Variational value	State	SRF	Variational value	State
1.00	1.08E-09	stable	1.25	8.61E-10	stable	1.50	7.58E-10	stable
1.05	1.04E-09	stable	1.30	7.48E-10	stable	1.55	2.21E-10	stable
1.10	1.01E-09	stable	1.35	1.01E-09	stable	1.56	1.69E-09	stable
1.15	8.09E-10	stable	1.40	7.83E-10	stable	1.57	1.70E-08	stable
1.20	1.04E-09	stable	1.45	5.25E-10	stable	1.58	−2.23E-08	unstable

**Table 12:** Results under different criteria.

Calculation conditions	Result of the paper [7]	Result of the FOS command	Result of the elastic energy criterion	Result of the variational criterion
Natural conditions	1.526	1.52	1.50	1.50
Anti-slide pile reinforcement	1.57	1.58	1.58	1.57

## 6 Discussions

As indicated in the previous context, the traditional failure criteria of the SRM (Criterion Ⅰ, Criterion Ⅱ, Criterion Ⅲ, and Criterion Ⅳ) exhibit certain inconveniences or limitations for tunnel portals. The proposed variational criterion demonstrates good applicability and generality.

For Criterion Ⅰ, the selection of monitoring point locations and displacement directions significantly affects the morphology of the displacement curves (Figs 3–5). Additionally, applying this criterion requires the arrangement of numerous monitoring points and the monitoring of displacements in three directions. It results in a substantial workload. For Criterion Ⅱ, the connectivity of the plastic zone is a qualitative concept. It lacks a quantitative standard. Besides, it necessitates manually inspecting the plastic zones of the model (Figs 6–8). It also involves considerable effort. Moreover, the criteria for plastic zone connectivity may vary across models with different situations. It leads to significant uncertainty. For Criterion Ⅲ, the non-convergence of the computational program lacks a clear mechanical explanation (Figs 9–11). For Criterion Ⅳ, the energy curve exhibits clear change patterns. At the moment of failure, the elastic zone energy value drops sharply, while the plastic zone energy value increases abruptly (Fig 12). This criterion shows promising application prospects but requires integrating and calculating the energy of each element. It involves extensive computations, high memory usage, and slow processing speed.

The proposed variational criterion addresses the aforementioned limitations to some extent. First, it eliminates the need for arranging monitoring points and determining displacement directions. It significantly reduces the workload. Second, it transforms the qualitative assessment of “failure” into a quantitative judgment based on the “sign of the variational value”. It provides a clearer standard. There is no need to examine the plastic zone distribution of the model. The application is brief. There is an applicability in different mesh densities, geometric dimensions, strength reduction factor intervals, mechanical parameters, and convergence criteria. It shows good generality. Third, this criterion is based on the principle of minimum potential energy. It provides a robust mechanical explanation for model failure. Finally, the variational value is calculated by simulating a single time step. It results in minimal computational effort, low memory usage, and fast processing speed. Additionally, the proposed variational criterion offers the following additional advantages. (1) The variational value incorporates stress and strain information from the entire model region. It makes the variational value a more comprehensive physical quantity. (2) The instability is determined by identifying the positive or negative of the variational value. It eliminates the need for manual failure judgment and reduces certain human errors.

As previously stated, the proposed variational criterion in this paper is currently applicable under the following conditions: the load is limited to gravity, the model boundaries are defined as non-working boundaries, and no constraints are imposed on the free surface of the model. Research indicates that, under these conditions, the applicability of the criterion is unaffected by either the shape of the external free surface or the internal mechanical characteristics of the model. This is evidenced by the slope-tunnel engineering example discussed herein (Figs 13 and 14, Tables 10–12), where the criterion remained valid despite an irregular free surface, heterogeneous soil layers, and the presence of an anti-slide pile. Furthermore, this conclusion is supported by additional calculations performed by the author using examples from the manual of Rocscience [34]. Specifically, by incorporating seismic forces, groundwater, and reinforcement materials into a slope numerical model, the variational criterion proved effective under these extreme load cases (Table 13). It highlights the strong application potential of the variational criterion. It is important to note that the proposed variational criterion is currently implemented primarily through numerical simulation. In practical engineering, it is necessary to establish an appropriate numerical model based on the engineering topography and geological conditions to realize the application of the variational criterion. Under more complex loading and boundary conditions, whether the variational criterion remains applicable still requires further study. In addition, further research is also needed to determine the corresponding practical physical quantity of the variational value, the formulas and methods required to obtain this physical quantity, and how to utilize the existing monitoring data.

**Table 13:** Results of examples from the manual of Rocscience.

Reference paper-slope stability verification manual	Number of the example	Loading conditions	Result of the reference paper	Result of the FOS command or self-programed command	Result of the elastic or plastic energy criterion	Result of the variational criterion
Part Ⅰ	3	Seismic force	0.97	0.98	1.00	0.97
Part Ⅰ	12	Groundwater	1.09	1.10	1.11	1.12
Part Ⅱ	48	Reinforcing material	1.05	1.05	1.06	1.06

Note: the elastic or plastic energy is calculated by referencing the method in papers [17,18], and it yields more ideal results.

## 7 Conclusions

In this paper, it examines the characteristics of traditional failure criteria and proposes a new criterion—the variational criterion for tunnel portals by using the SRM. Several ideal and engineering examples are analyzed to verify the applicability of the proposed variational criterion. The main conclusions are as follows:

(1) Criterion Ⅰ is cumbersome to apply and involves a significant workload. Criterion Ⅱ lacks quantitative and clear indicators. Criterion Ⅲ lacks a mechanical explanation. Criterion Ⅳcan effectively reflect the failure mechanism of the model, but the computational effort is relatively large.
(2) The proposed variational criterion addresses the shortcomings of traditional failure criteria. It provides a comprehensive indicator—the variational value. It adopts a more explicit discrimination method—judging the sign of the variational value. In this way, it is with low computational effort and strong objectivity, and offers a theoretical explanation for the model failure. This criterion can provide a new reference for stability identification in tunnel portals.
(3) For an ideal tunnel portal with a regular free surface, homogeneous soil layers, and no anti-slide piles, the proposed variational criterion remains applicable. This applicability is unaffected by mesh densities, geometric parameters, strength factor intervals, mechanical parameters, or convergence criteria. For an engineered slope-tunnel system characterized by an irregular free surface, heterogeneous soil layers, and the presence of anti-slide piles, the proposed variational criterion continues to demonstrate strong applicability. Preliminary verification shows that the proposed variational criterion is applicable to extreme conditions involving seismic force, groundwater, and reinforcing materials in slopes.
(4) Based on the existing findings, the engineering application steps of the proposed variational criterion are currently as follows: (i) an appropriate engineering numerical model is established in FLAC3D; (ii) the variational value under each SRF is calculated using the written program; (iii) the stability of the model is identified by judging the sign of the variational value, and the FOS is determined. The engineering applicability boundaries of this criterion are also currently as follows: (a) the action of gravity, seismic force, groundwater, or reinforcing materials can be considered; (b) the boundaries used to constrain the model are non-working boundaries; (c) no requirements are imposed on the shape of the free surface of the model.
