# Correction: A new criterion for defining tunnel portal failure using the strength reduction method

**DOI:** 10.1371/journal.pone.0351737

**Published:** 2026-06-12

**Authors:** Chengya Hua, Hongzhou Zhang, Chenguang Song, Leihua Yao

The Funding statement for this article is incorrect. The correct Funding statement is as follows: This study was financially supported by the Science Research Project of Hebei Education Department (https://kygl.hdvtc.edu.cn) in the form of a grant (ZC2025097) received by CH. This study was also financially supported by the National Natural Science Foundation of China (https://www.nsfc.gov.cn) in the form of a grant (41807228) received by LY.
